# Ethylbenzene Removal by Carbon Nanotubes from Aqueous Solution

**DOI:** 10.1155/2012/817187

**Published:** 2011-12-05

**Authors:** Bijan Bina, Hamidreza Pourzamani, Alimorad Rashidi, Mohammad Mehdi Amin

**Affiliations:** ^1^Environment Research Center, Isfahan University of Medical Sciences, Isfahan, Iran; ^2^Environment Research Center and Department of Environmental Health Engineering, School of Health, Isfahan University of Medical Sciences, Isfahan, Iran; ^3^Gas Division, Research Institute of Petroleum Industry (RIPI), Tehran, Iran

## Abstract

The removal of ethylbenzene (E) from aqueous solution by multiwalled, single-walled, and hybrid carbon nanotubes (MWCNTs, SWCNTs, and HCNTs) was evaluated for a nanomaterial dose of 1 g/L, concentration of 10–100 mg/L, and pH 7. The equilibrium amount removed by SWCNTs (E: 9.98 mg/g) was higher than by MWCNTs and HCNTs. Ethylbenzene has a higher adsorption tendency on CNTs, so that more than 98% of it adsorbed in first 14 min, which is related to the low water solubility and the high molecular weight. The SWCNTs performed better for ethylbenzene sorption than the HCNTs and MWCNTs. Isotherms study indicates that the BET isotherm expression provides the best fit for ethylbenzene sorption by SWCNTs. Carbon nanotubes, specially SWCNTs, are efficient and rapid adsorbents for ethylbenzene which possess good potential applications to maintain high-quality water. Therefore, it could be used for cleaning up environmental pollution to prevent ethylbenzene borne diseases.

## 1. Introduction

Ethylbenzene (aromatic organic compound) is important on material in the chemical process industries. This material is usually used as raw material in numerous chemical productions and also often as solvent in a wide variety of manufacturing processes [1].

Ethylbenzene is widely used in industry as solvents for organic synthesis, equipment cleaning, and other downstream processing purposes. They are present in refinery and chemical industry effluents. The ethylbenzene is frequently found in groundwater because of leaks in underground storage tanks and pipelines, improper waste disposal practices, inadvertent spills, and leaching from landfills [2]. This pollutant has been found to cause many serious health side effects to humans (e.g., skin and sensory irritation, central nervous system depression, respiratory problems, leukemia, cancer, and disturbance of the kidney, liver, and blood systems) [3].

Ethylbenzene removal from groundwater has been widely studied, and several processes have been successfully applied. A considerable effort has been dedicated in the past years concerning the removal of ethylbenzene from water and wastewater, several methods have been proposed and developed, and the most extensively used is adsorption process.

Carbon nanotubes (CNTs) have attracted great interest because of their unique chemical structure and intriguing physical properties [4]. The large adsorption capacity of CNTs for organic pollutant is primarily due to their pores structure and the existence of a wide spectrum of surface functional groups. The adsorption mechanism of ethylbenzene on CNTs is mainly attributed to the *π*-*π* electron donor-acceptor interaction between the aromatic ring of ethylbenzene and the surface carboxylic groups of CNTs [5].

The adsorption of ethylbenzene on Pt and on epitaxial FeO and Fe_3_O_4_ films is studied by Ranke and Weiss [6]. Lu et al. used surface modification of carbon nanotubes to enhance ethylbenzene adsorption from aqueous solutions. The NaOCl-oxidized CNTs have superior adsorption performance toward ethylbenzene with many types of carbon and silica adsorbents reported in the literature [5]. Su et al. employed multiwalled carbon nanotubes (MWCNTs) that were oxidized by sodium hypochlorite (NaOCl) solution to enhance the adsorption of ethylbenzene in an aqueous solution [7]. Aivalioti et al. studied the removal of ethylbenzene from aqueous solutions by raw (DR) and thermally modified diatomite [3].

These studies indicate that CNTs have high affinity with both organic and inorganic chemicals. Literature also showed the potential for developing carbon nanotube technologies for treating ethylbenzene in water. It is commonly discharged from many kinds of industrial activities and frequently encountered in the groundwater of gasoline-contaminated sites [3–7].

The present study aimed to determine the removal efficiency for ethylbenzene using single-walled and multiwalled carbon nanotubes and hybrid carbon nanotubes and to rank their ethylbenzene removal abilities. The contribution of this study is the evaluation of using hybrid CNTs in ethylbenzene removal that was not found in the literature.

## 2. Materials and Methods

### 2.1. Materials

The chemical tested in this study was ethylbenzene (Merck, purity: 99.7%). A stock solution of approximately 100 mg/L of ethylbenzene was prepared by dissolving appropriate amounts of substance in a standard solution that contained 100 mg/L of ethylbenzene in deionized H_2_O. The mixture was mixed thoroughly by using an ultrasonic bath (BANDELIN Sonorex Digtec) for 60 min. Then, it was stirred continuously for 24 h at 25°C. After shaking, the solution was put in the ultrasonic bath again for 30 min and was used to prepare the initial ethylbenzene solution with a 10–100 mg/L concentration. Finally, standard series and samples were prepared using deionized H_2_O to achieve the desired concentrations.

### 2.2. Experimental Conditions

Batch adsorption experiments were conducted using 110 mL glass bottles with the addition of 100 mg of adsorbents and 100 mL of ethylbenzene solution at an initial concentration (*C*_0_) of 10 mg/L. It was chosen to be representative of low ethylbenzene level in water polluted with gasoline. The glass bottles were sealed with 20 mm stoppers. The headspace within each beaker was minimized to exclude any contaminant volatilization phenomena. The glass bottles from the batch experiments were placed on a shaker (Orbital Shaker Model OS625) and were stirred at 240 rpm at room temperature for 10 minutes. The solution samples were then allowed to settle for 2 min. Finally, the ethylbenzene concentration in the liquid phase was determined using gas chromatography with detector of mass spectrometry (GC/MS). All of the experiments were repeated three times, and only the mean values were reported. Blank experiments, without the addition of adsorbents, were also conducted to ensure that the decrease in ethylbenzene concentration was not due to adsorption on the wall of the glass bottle or volatilization. The pH_in_ was adjusted to neutral using 0.05 M HCl or 0.05 M NaOH. The amounts of adsorbed ethylbenzene on the adsorbents (*q*_*e*_, mg/g) were calculated as follows:

(1) $$\begin{array}{c} q_{e}=\left(C_{0}-C_{t}\right)\times \frac{V}{m}, \end{array}$$

where *C*_0_ and *C*_*t*_ (mg/L) are the ethylbenzene concentrations at the beginning and after a certain period of time, *V* is the initial solution volume (Li), and *m* is the adsorbent weight (g).

### 2.3. Chemical Analysis

An Agilent Technologies system consisting of a 5975C inert MSD with a triple-axis detector equipped with a 7890A gas chromatograph with a split/splitless injector was used for ethylbenzene measurements. A fused silica column, HP-5 ms (5% phenyl-95% dimethylpolysiloxane; 30 m × 0.25 mm I.D, 0.25 *μ*m), was employed with helium (purity 99.995%) as the carrier gas at a flow rate of 1 mL/min. The column temperature was programmed as follows: 36°C for 2 min, increasing to 140°C at 10°C/min and holding for 6 min. The injector port was maintained at 210°C, and a 1 mL volume of headspace was injected in splitless mode (2.0 min). The effluent from the GC column was transferred via a transfer line held at 280°C and fed into a 70 eV electron impact ionization source held at 280°C. The data were acquired and processed by the data analysis software.

Static headspace analysis was performed using a CTC PAL-Combi PAL headspace sampler. The experimental parameters of the headspace sampler were as follows: incubation time, 25 min; incubation temperature, 70°C; sample loop volume, 1 mL; syringe/transfer line temperature, 110°C; flash time, 2 min with N_2_; loop fill time, 0.03 min; injection time, 1 min; and sample volume, 10 mL in 20 mL vials. No NaCl was added to the samples.

The pH measurements were made with a pH meter (EUTECH, 1500).

### 2.4. Adsorbents

**Figure 1:**
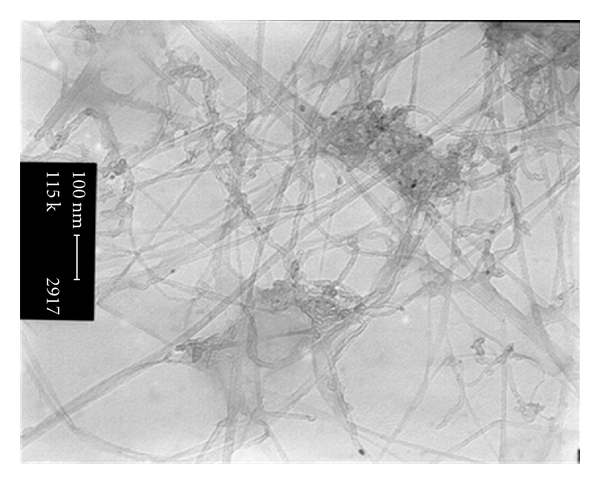
TEM image of SWCNT.

**Figure 2:**
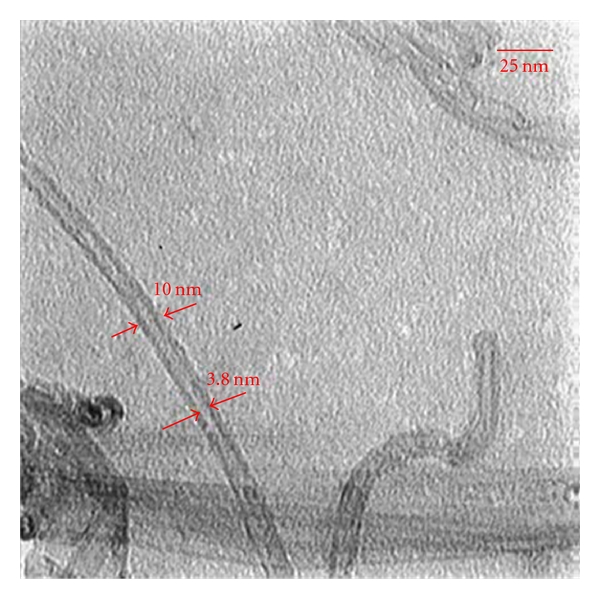
TEM image of MWCNT.

**Figure 3:**
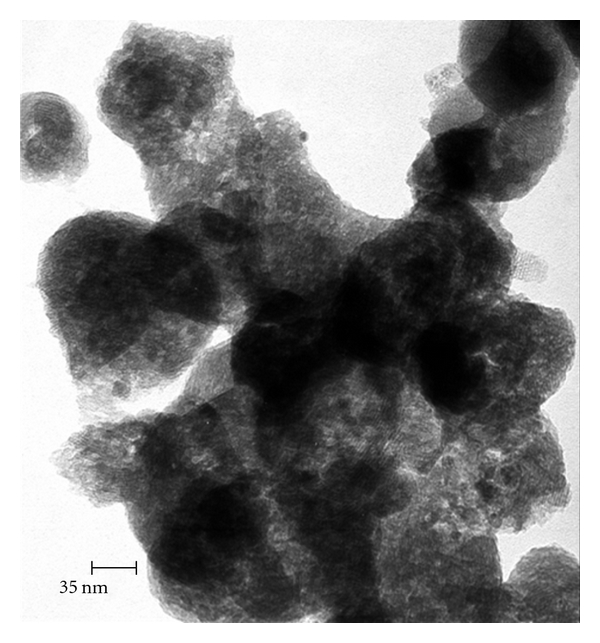
TEM image of HCNT.

During the experimental procedure, three different nanomaterials were tested: (1) single-walled carbon nanotubes (SWCNTs), (2) multi-wall carbon nanotubes (MWCNTs), and (3) hybrid carbon nanotubes (HCNTs). The SWCNTs with 1-2 nm diameter (Figure 1), MWCNTs with 10 nm diameter (Figure 2), and HCNTs were a hybrid of MWCNTs and silica (Figure 3) to open the tubes of MWCNT as a sheet instead of tube. These adsorbents were purchased from the Iranian Research Institute of the Petroleum Industry.

### 2.5. Analysis of Data

For the data analysis, design of experiments (DOE) software (Design Expert 6) was used. In this software, the analysis was done with a general factorial plan. Isotherm Fitting Tool (ISOFIT) is a software program that fits isotherm parameters to experimental data via the minimization of a weighted sum of squared error (WSSE) objective function. ISOFIT supports a number of isotherms, including (1) Brunauer-Emmett-Teller (BET), (2) Freundlich, (3) Freundlich with Linear Partitioning (F-P), (4) Generalized Langmuir-Freundlich (GLF), (5) Langmuir, (6) Langmuir with Linear Partitioning (L-P), (7) Linear, (8) Polanyi, (9) Polanyi with Linear Partitioning (P-P), and (10) Toth. Observation weights are ideally assigned according to individual estimates of measurement error, such that *w*_*i*_ = 1/*sd*⁡_*i*_, where *sd*⁡_*i*_ is the standard deviation of the *i*th measurement.

### 2.6. Recycling Method

The reversibility of the sorbents that were used for ethylbenzene removal from aqueous solution was evaluated via 2 successive adsorption cycles followed by 2 successive desorption cycles. Recycling was also conducted at 105 ± 2°C and 24 h in an oven (Memmert D-91126, Schwabach FRG). All samples were replicated at least in triplicate.

## 3. Results and Discussion

### 3.1. Adsorption Performance

Table 1 shows the ethylbenzene removal percent for MWCNTs, SWCNTs, and HCNTs under an initial ethylbenzene concentration of 10 mg/L, an adsorbent concentration of nanomaterial of 1000 mg/L, contact time of 10 min, and shaking at 240 rpm.

Based on the DOE analysis, there were differences between MWCNTs, SWCNTs, and HCNTs for ethylbenzene removal (values of “Prob > |*t*|” less than 0.05).

**Figure 4:**
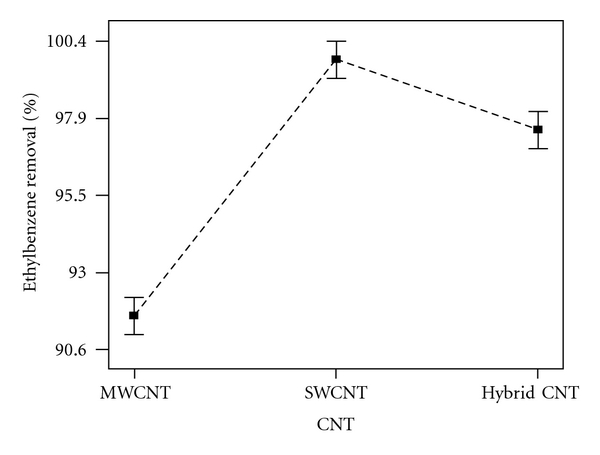
Design expert plot for MWCNT, SWCNT, and HCNT in ethylbenzene removal with a *C*_0_ of 10 mg/L.

Figure 4 shows the ethylbenzene removal by MWCNTs, SWCNTs, and HCNTs and a comparison between them. SWCNTs were better than HCNTs and MWCNTs, and also HCNTs were better than MWCNTs at removing ethylbenzene.

**Figure 5:**
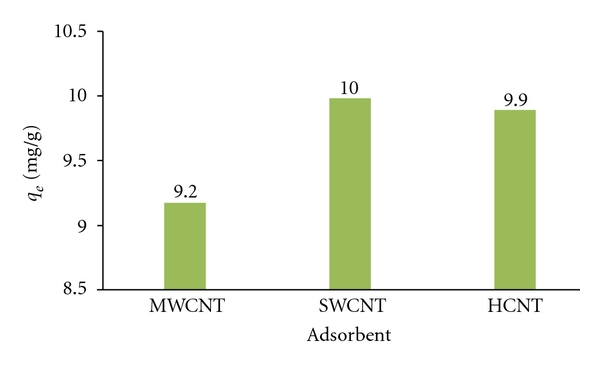
Equilibrium amount of ethylbenzene adsorbed on CNTs with a *C*_0_ of 10 mg/L.

Figure 5 indicates the equilibrium amounts of ethylbenzene adsorbed on MWCNTs, SWCNTs, and HCNTs (*q*_*e*_) with a *C*_0_ of 10 mg/L and contact time of 10 min.

The results from this study showed that the *q*_*e*_ for SWCNTs is higher than for HCNTs and MWCNTs. With a *C*_0_ of 10 mg/L, the SWCNTs showed the greatest *q*_*e*_ (ethylbenzene: 9.98 mg/g). The equilibrium amount (*q*_*e*_) for ethylbenzene adsorption sequence is SWCNTs > HCNTs > MWCNTs.

Equilibrium amount from Lu et al. study for surface modification of carbon nanotubes *q*_*e*_ for ethylbenzene obtained 180 mg/g for a *C*_0_ of 60 mg/L [5]. And in study of Aivalioti et al., *q*_*e*_ for ethylbenzene adsorbed by raw diatomite soil and thermally modified diatomite were 0.3 and 0.6 mg/g, respectively [3]. The equilibrium results from Su et al. study showed that the *q*_*e*_ of ethylbenzene adsorption (at a *C*_0_ of 200 mg/L, contact time of 240 min, and 600 mg/L of adsorbent concentration) by raw CNT and modified CNT by NaOCl were 255 and 274 mg/g, respectively [7].

The results imply the presence of chemically inherited groups that lead to direction of the affinity for ethylbenzene removal, irrespective of the texture characteristics. This indicates that the adsorption of ethylbenzene on CNTs is dependent on the surface chemical nature and the porosity characteristics. Similar findings have been reported in the literature for the adsorption of ethylbenzene on activated carbon [8] and multiwalled carbon nanotubes (MWCNTs) [5, 7].

As shown in Figure 3 used of silica with MWCNT as HCNT, silica cause opens the tubes of MWCNTs and produce sheet of carbon nanotubes, which have more area than MWCNTs for ethylbenzene adsorption. It is response for more removal of ethylbenzene by HCNTs than MWCNTs.

Furthermore, the electrostatic interaction between the ethylbenzene molecules and the SWCNT surface may also explain the observation of high ethylbenzene adsorption via the single-walled CNTs. Because the ethylbenzene molecules are positively charged [5], the adsorption of ethylbenzene is thus favored for adsorbents with a negative surface charge. This results in more electrostatic attraction and thus leads to a higher ethylbenzene adsorption.

Under analogous conditions, the present SWCNTs and HCNTs show better performance for ethylbenzene adsorption than do other adsorbents. This suggests that the SWCNTs and HCNTs are efficient ethylbenzene adsorbents. Because the costs of commercially available HCNTs are continuously decreasing, it may be possible to utilize these novel nanomaterials for ethylbenzene removal in water and wastewater treatment in the near future.

### 3.2. CNTs Recycling

Repeated availability is an important factor for an advanced adsorbent. Such adsorbent not only possesses higher adsorption capability, but also should show better desorption property, which will significantly reduce the overall cost for the adsorbent.

**Figure 6:**
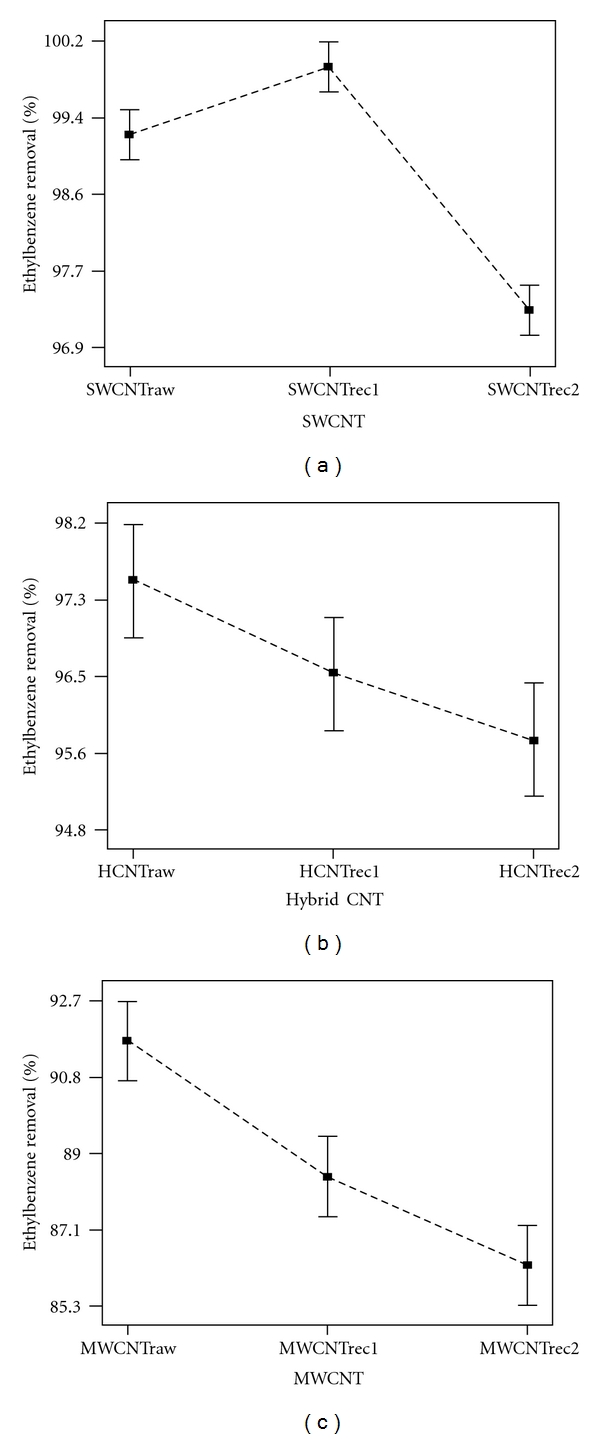
Design expert plot for raw and recycled CNTs in ethylbenzene removal: (a): SWCNTs, (b): HCNTs, and (c): MWCNTs.

Although CNTs show more ethylbenzene sorption capacities form aqueous solution, very high unit cost currently restricts their potential use in water and wastewater treatment. Thus, testing the reversibility of sorbents used for ethylbenzene removal is required in order to reduce their replacement cost. For this purpose as a part of the study, probability for used SWCNTs, MWCNTs, and HCNTs recycling was investigated. Table 2 shows the ethylbenzene removal percent by MWCNTs, SWCNTs, and HCNTs that were recycled in the first cycle (MWCNTrec1, SWCNTrec1, and HCNTrec1) and MWCNTs, SWCNTs, and HCNTs that were recycled in the second cycle (MWCNTrec2, SWCNTrec2, and HCNTrec2) under initial ethylbenzene concentration of 10 mg/L, nanomaterial concentration of 1000 mg/L, contact time of 10 min, and shaking at 240 rpm.  Figure 6 compares raw SWCNTs, HCNTs, and MWCNTs with their recycling in cycles of 1 and 2.

It is apparent that CNTs can be reused for the removal of ethylbenzene through a large number of water and wastewater treatment and regeneration cycles. The presence of metal catalysts in raw SWCNTs that could be retained through the chemical process to functionalize of SWCNTs may be removed due to heating and cause better adsorption performance for recycled SWCNTs than raw SWCNTs. Also the structure and nature of carbon surface were changed after thermal treatment including the increase in graphitized structure and the decrease in surface functional groups and negative charges [9]. These specifications of SWCNTs cause more adsorption of ethylbenzene.

Results show that the ethylbenzene adsorbed by the SWCNTs, MWCNTs, and HCNTs could be easily desorbed by temperature, and thereby they can be employed repeatedly in water and wastewater management. This is the key factor for whether a novel but expensive sorbent can be accepted by the field or not. It is expected that the unit cost of CNTs can be further reduced in the future by recycling heat processes. So; the SWCNTs, HCNTs, and MWCNTs appear possibly cost effective ethylbenzene sorbents in water and wastewater treatment.

As CNTs and carbonaceous materials such as black carbon, coal, and kerogen in soils/sediments that are composed of almost the same element, desorption difference may be due to their distinct geometric structure. The carbonaceous materials exhibit a high degree of porosity and extended interparticulate surface area, whereas CNTs are one dimensional hollow nanosize tubes as well as aggregates. CNTs easily adhere to each other and form bundles due to strong Van der Waals interactions. The adsorption sites are therefore defined for the entire bundles instead of individual nanotubes. There are four possible groups of adsorption sites on bundles, including interior of individual tubes, interstitial channels between nanotubes, external groove sites, and the outer surface sites of individual tubes on the peripheral surface of the bundles. The interior of individual tubes is only available in open-ended tubes; the interstitial channels are applied for large tube diameters, while grooves and the external surface are of most importance for adsorption [4]. Therefore, it is inferred that most of the ethylbenzene is located on the external adsorption sites. Moreover, CNTs cannot form closed interstitial spaces in their aggregates. Hence, ethylbenzene adsorbed release due to temperature.

The sorbent weight loss was neglected in the recycling processes. The weight loss can be attributed to the evaporation of adsorbed water and the elimination of carboxylic groups and hydroxyl groups on the CNT wall [5].

### 3.3. Isotherm Study

The adsorption equilibrium data of ethylbenzene on SWCNTs, HCNTs, and MWCNTs samples were fitted by several well-known isotherm models to assess their efficacies. In this study, ISOFIT was applied to involving the adsorption of ethylbenzene with initial concentration of 10–100 mg/L (10, 20, 30, 40, 50, 60, 70, 80, 90, and 100 mg/L) by SWCNTs, HCNTs, and MWCNTs. Water solubility (*S*_*w*_) of ethylbenzene was estimated 152 mg/L at pH 7. Numerous studies have considered one or more of the supported isotherms in the context of a water-wastewater system. The dual-mode isotherms reflect recently developed models for the sorption of hydrophobic organic solutes [10].

To characterize parameter uncertainty, ISOFIT reports parameter correlation coefficients and 95% linear confidence intervals for each isotherm parameter. The correlation coefficient (CORij) between parameters Xi and Xj is a measure of the linear dependence between the two parameters; values range from −1 to *‏*1, with a value of zero (0) indicating no correlation.

Isotherm expressions are important for describing the partitioning of contaminants in environmental systems.  Table 3 summarizes some of the diagnostic statistics computed by ISOFIT and reported in the output file for ethylbenzene adsorption by CNTs.

**Figure 7:**
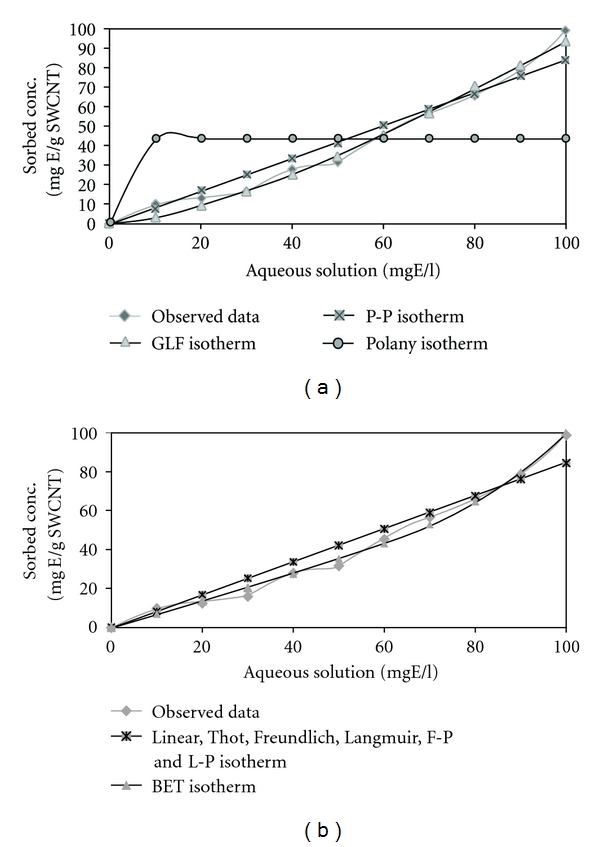
Plots of fitted isotherms and observed data of ethylbenzene adsorption by SWCNTs: (a) Toth, P-P, GLF, and Polanyi, (b) linear, Freundlich, Langmuir, F-P, L-P, and BET.

Figure 7 contains plots of the fitted isotherms for ethylbenzene adsorption by SWCNTs, (organized into visually indistinguishable groups) along with the observed data points.

**Figure 8:**
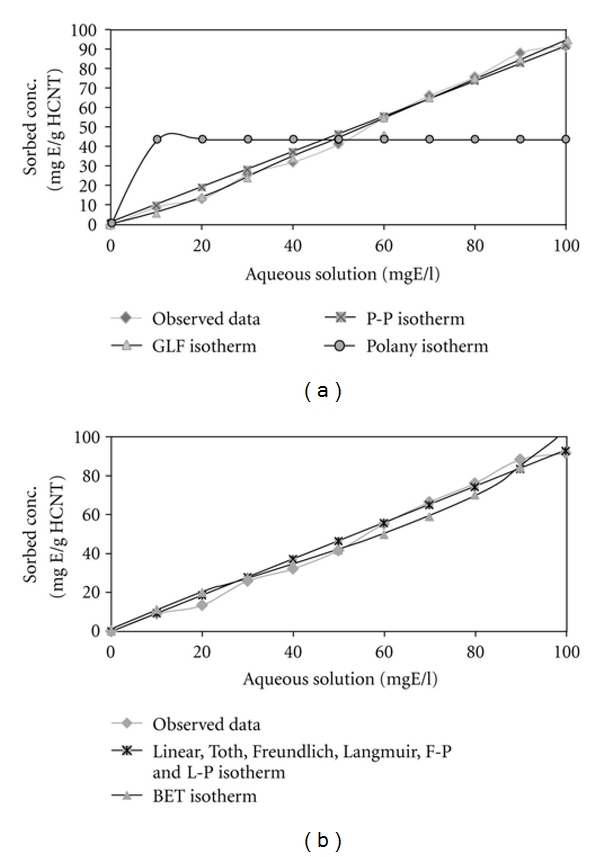
Plots of fitted isotherms and observed data of ethylbenzene adsorption by HCNTs: (a) Toth, P-P, GLF, and Polanyi, (b) linear, Freundlich, Langmuir, F-P, L-P, and BET.

Figure 8 contains plots of the fitted isotherms for ethylbenzene adsorption by HCNTs, (organized into visually indistinguishable groups) along with the observed data points.

**Figure 9:**
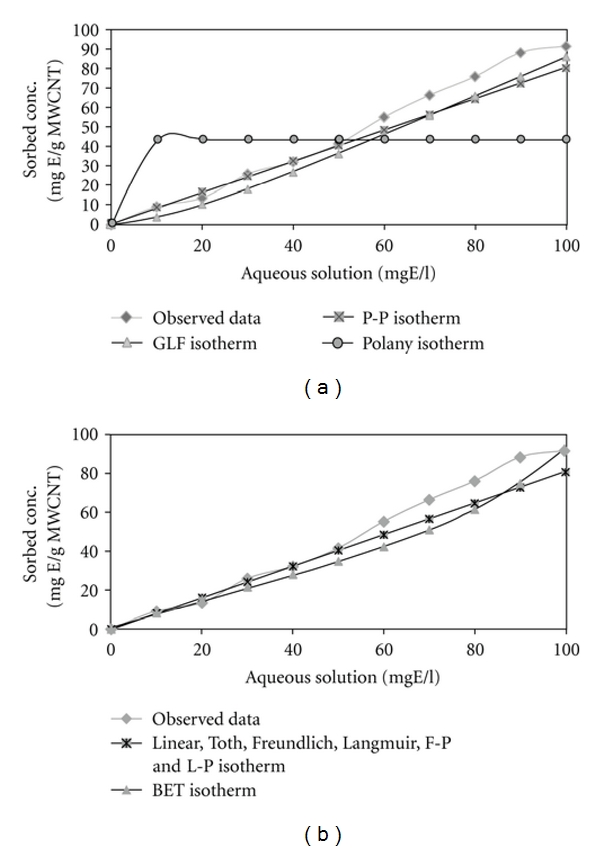
Plots of fitted isotherms and observed data of ethylbenzene adsorption by MWCNTs: (a) Toth, P-P, GLF, and Polanyi, (b) linear, Freundlich, Langmuir, F-P, L-P, and BET1.

Figure 9 contains plots of the fitted isotherms for ethylbenzene adsorption by HCNTs, (organized into visually indistinguishable groups) along with the observed data points.

The AICc values indicate that the BET isotherm expression provides the best fit of ethylbenzene adsorption by SWCNTs, GLF isotherm expression provides the best fit of ethylbenzene adsorption by HCNTs, and GLF isotherm is the best fit for ethylbenzene adsorption by MWCNTs.

In this study, the results showed that ISOFIT provided superior fits in ethylbenzene removal by SWCNTs, for BET, GLF, and Polanyi isotherms and for ethylbenzene removal by HCNTs and MWCNT and provided superior fits for all isotherms. ISOFIT was used by Shawn Matott to the adsorption of zinc by ferrihydrite. Results indicate that ISOFIT produced equivalent or better fits values. In particular, ISOFIT provided superior fits for the GLF, Toth, Polanyi, and Polanyi-Partition isotherms [10]. The adsorption process of atrazine on CNTs studyed by Yan et al. shows the adsorption equilibrium isotherms were nonlinear and were fitted by Freundlich, Langmuir, and Polanyi-Manes models. It was found that the Polanyi-Manes model described the adsorption process better than other two isotherm models [4].

Wibowo et al. studied the adsorption of benzene and toluene from aqueous solutions onto activated carbon; their study shows that the Langmuir equation can describe the experimental data fairly well than Freundlich [1].

In the adsorption mechanism of aromatic compounds in liquid phase on SWCNT, there are two main types of interactions, including electrostatic and dispersive. The functional group linked to the adsorptive aromatic ring can activate or deactivate it that removes its electronic charge. Electron with drawing groups on an aromatic ring create a partial positive charge in the ring, while deactivating groups produce the opposite effect, creating a partial negative charge [1].

Here, ethylbenzene is in the molecular form in the aqueous solution; in this case, dispersive interactions are predominant, mainly because of the attraction between the *π* orbital on the SWCNT basal planes and the electronic density in the benzene and toluene aromatic rings (*π*-*π* interactions) [1].

The limitations of this study were using constant condition for ethylbenzene removal. Somewhat separation of nanotubes from supernatant was also difficult. Carbon nanotubes are in the nano of one dimension. Also, to provide a well adsorption; ethylbenzene should penetrate into the tubes. This causes slow absorption, and increasing the contact time is required. Similar findings have been reported in the literature for the adsorption of ethylbenzene by multiwalled carbon nanotubes (MWCNTs) [7]. But, in the hybrid nanotubes sheets, these tubes are opened and contact surfaces are more easily available.

## 4. Conclusion

We concluded that SWCNTs showed a higher adsorption capacity for ethylbenzene removal than MWCNTs and HCNTs. Also between MWCNT and HCNT, HCNT shows better performance for ethylbenzene removal because of sheet shape produced by silica. It appears that ethylbenzene is the component with higher adsorption tendency on CNTs. The equilibrium amount (*q*_*e*_) sequence is SWCNTs > HCNTs > MWCNTs.

Results of CNTs recycling show that the ethylbenzene adsorbed by the SWCNTs, MWCNTs, and HCNTs could be easily desorbed by temperature, and thereby they can be employed repeatedly in water and wastewater management.

ISOFIT provided superior fits for the BET isotherm for ethylbenzene removal by SWCNTs, and GLF fits for ethylbenzene removal by HCNTs and MWCNTs.

More research works on the toxicity of CNTs and CNT-related materials are needed before practical use of CNTs in water and wastewater treatment because it can be realized.

## Figures and Tables

**Table 1:** Ethylbenzene removal by MWCNT, SWCNT, and HCNT at *C*_0_ = 10 mg/L and contact time of 10 min.

Absorbent	Ethylbenzene	
	*C*_*t*_ (mg/L)	Removal percent (%)
MWCNT	0.23	91.7
SWCNT	0.03	99.5
HCNT	0.04	97.6

**Table 2:** Ethylbenzene removal by raw and recycled MWCNTs, SWCNTs, and HCNTs at *C*_0_ = 10 mg/L and contact time of 10 min.

Adsorbent	Ethylbenzene	
	*C*_*t*_ (mg/L)	Removal percent (%)
MWCNTrec1	1.16	88.5
SWCNTrec1	0.01	99.8
HCNTrec1	0.35	96.5
MWCNTrec2	1.37	86.3
SWCNTrec2	0.23	97.7
HCNTrec2	0.43	95.7

**Table 3:** Summary of selected diagnostics for ethylbenzene adsorbed by CNTs.

Adsorbents	Selected diagnostics	Isotherms									
		GLF	Toth	Linear	Langmuir	F-P	L-P	Freundlich	P-P	BET	Polanyi
SWCNTs	AICc	34.4	41.5	41.5	41.5	41.5	41.5	44.7	45.5	24.5	72.8
	*R*_*y*_^2^	0.988	0.962	0.962	0.962	0.962	0.962	0.962	0.962	0.992	0.000
	*R*_*N*_^2^	0.934	0.892	0.892	0.892	0.892	0.892	0.892	0.888	0.966	0.947
	*M* ^2^	30	1 × 10^−9^	3 × 10^−9^	2 × 10^−10^	3 × 10^−10^	4 × 10^−10^	7.3	5 × 10^−1^	2 × 10^−2^	9 × 10^5^
	Linearity assessment	Non-linear	Linear	Linear	Linear	Linear	Linear	Non- linear	Non-linear	Linear	Non-linear

HCNTs	AICc	25.9	26.3	26.3	26.3	26.3	29.5	29.5	31.3	40.7	72.8
	*R*_*y*_^2^	0.994	0.992	0.992	0.992	0.992	0.992	0.992	0.992	0.960	0.000
	*R*_*N*_^2^	0.946	0.942	0.942	0.942	0.942	0.942	0.942	0.952	0.945	0.957
	*M* ^2^	83	6 × 10^−10^	1 × 10^−9^	8 × 10^−10^	6 × 10^−10^	3 × 10^2^	20	5 × 10^−1^	10	2 × 10^6^
	Linearity assessment	Non-linear	Linear	Linear	Linear	Linear	Non-linear	Non- linear	Non-linear	Linear	Non-linear

MWCNTs	AICc	30.7	34.8	34.8	34.8	34.8	34.8	38.6	39.3	37.6	68.1
	*R*_*y*_^2^	0.989	0.980	0.980	0.980	0.980	0.980	0.979	0.980	0.965	—
	*R*_*N*_^2^	0.931	0.914	0.914	0.914	0.914	0.914	0.914	0.918	0.956	0.944
	*M* ^2^	39	1 × 10^−9^	2 × 10^−9^	2 × 10^−9^	2 × 10^−9^	2 × 10^−9^	11	5 × 10^−1^	11	3 × 10^−9^
	Linearity assessment	Linear	Linear	Linear	Linear	Linear	Linear	Non- linear	Non-linear	Linear	Linear

AICc: Multimodel ranking, *R*_*y*_^2^: Correlation between measured and simulated observation, *R*_*N*_^2^: Correlation between residual and normality, *M* ^2^: Linssen measure of nonlinearity.
